# Supplementary material for: Russian Doll Genes and Complex Chromosome Rearrangements in Oxytricha trifallax
Source: G3 (Bethesda). 2018 Mar 15;8(5):1669–74. doi: 10.1534/g3.118.200176 (PMC5940158; doi:10.1534/g3.118.200176)
Supplement: Supplementary file 1 [file 1669FileS1.pdf]

contig5288.0 & contig20275.0.1

MDSs found in MIC locus ctg67399

$\overline{115}$ – $\overline{131\text{-}116}$ – $\overline{114\text{-}63}$ – 17-24 – 1-16 – $\overline{47\text{-}25}$ – 48-62 – $\overline{117}$ – $\overline{133\text{-}118}$ – $\overline{116\text{-}66}$ – 16-24 – 1-15 – $\overline{49\text{-}25}$ – 50-65

Linear representation of MDSs in the MIC locus (not to scale). Red: contig5288.0; blue contig20275.0.1.

Pointer list: [6, 5, 6, 5, 4, 1, 2, 1, 3, 2, 3, 4] (for both)

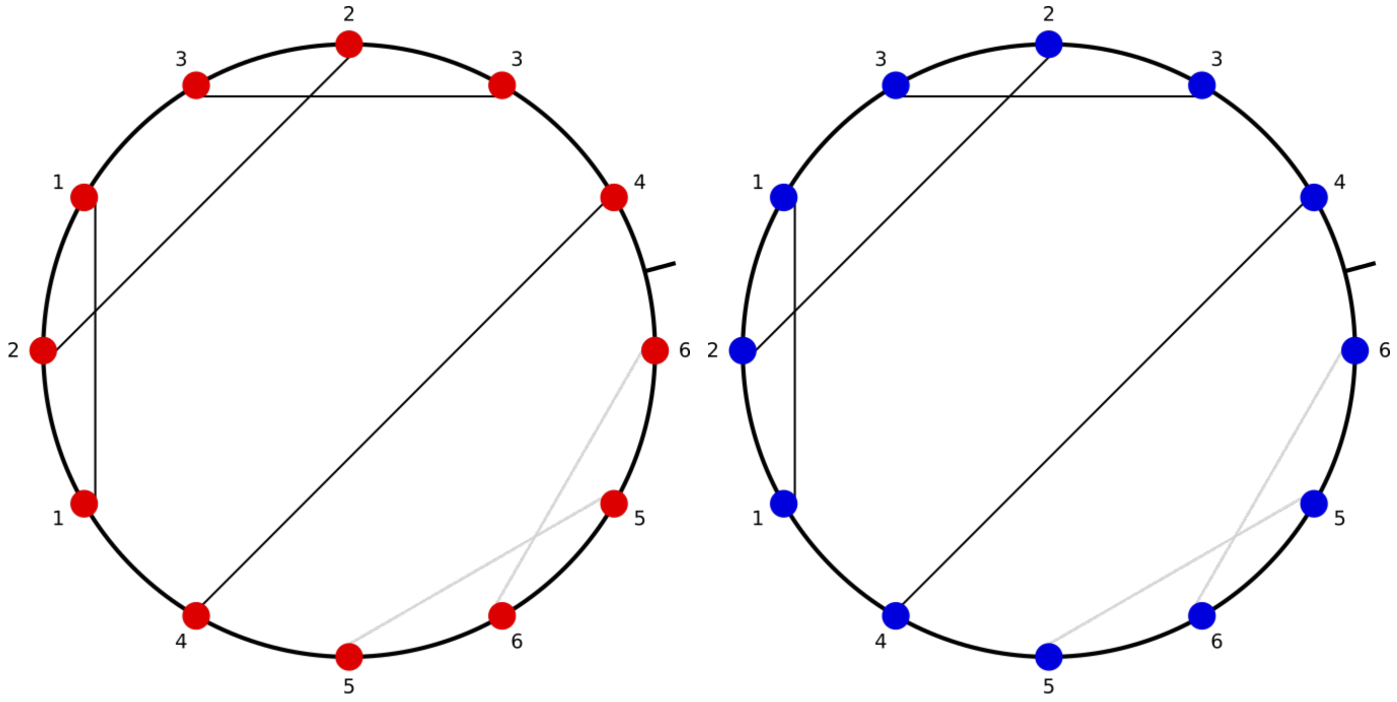

Chord diagrams for contig5288.0 (red, left) and contig20275.0.1 (blue, right). Black tick mark indicates the start of the scrambled pointer list (reading clockwise). Grey lines indicate pointers in repeat/return words. Black lines indicate the pointers remaining after iterative removal of odd-even patterns (repeat and return words).

contig2629.0

MDSs found in MIC locus ctg89774

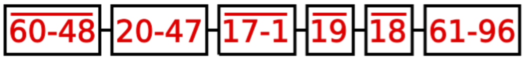

Linear representation of MDSs in the MIC locus.

Pointer list: [4, 3, 2, 3, 1, 2, 1, 4]

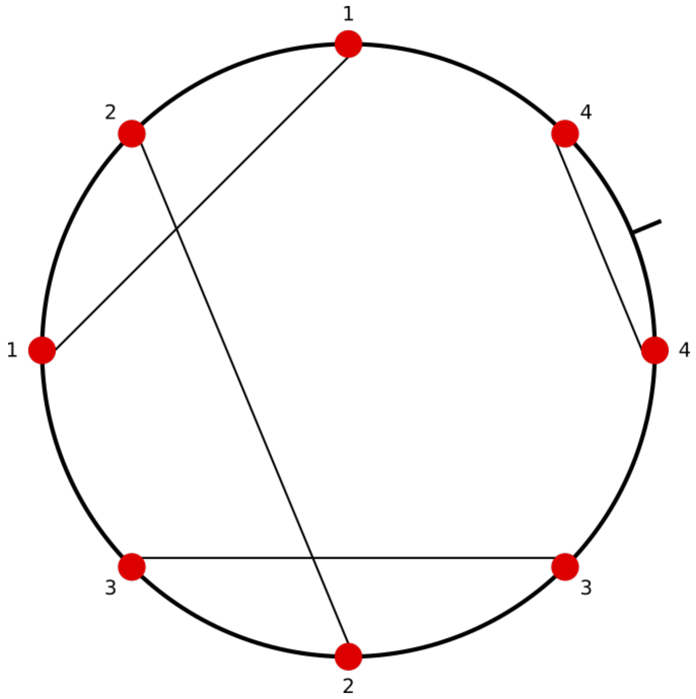

Chord diagram for contig2629.0
Black tick mark indicates the start of the scrambled pointer list.
Black lines indicate scrambled pointers.

contig6325.0.0

MDSs found in MIC locus ctg90297

41-47 odd - 50 - 52 - 55 - 57 - 59 - 39 - 35 - 33 - 29 - 27 - 24 - 22 - 19-5 odd - 2 - 1 - 4-10 even - 21 - 23 - 25 - - - -

- - - - - 31 - 34-40 even - 58 - 54 - 49 - 46 - 42 - 44 - 48 - 51 - 53 - 56 - 60 - 61 - 37 - 32-26 even - 20-12 even - 3

Linear representation of MDSs in the MIC locus.

Pointer list: [40, 41, 42, 43, 44, 45, 46, 47, 49, 50, 51, 52, 54, 55, 56, 57, 58, 59, 39, 38, 35, 34, 33, 32, 29, 28, 27, 26, 24, 23, 22, 21, 19, 18, 17, 16, 15, 14, 13, 12, 11, 10, 9, 8, 7, 6, 5, 4, 2, 1, 1, 3, 4, 5, 6, 7, 8, 9, 10, 20, 21, 22, 23, 24, 25, 30, 31, 33, 34, 35, 36, 37, 38, 39, 40, 58, 57, 54, 53, 49, 48, 46, 45, 42, 41, 43, 44, 47, 48, 50, 51, 52, 53, 55, 56, 59, 37, 36, 32, 31, 30, 29, 28, 27, 26, 25, 20, 19, 18, 17, 16, 15, 14, 13, 12, 11, 3, 2]

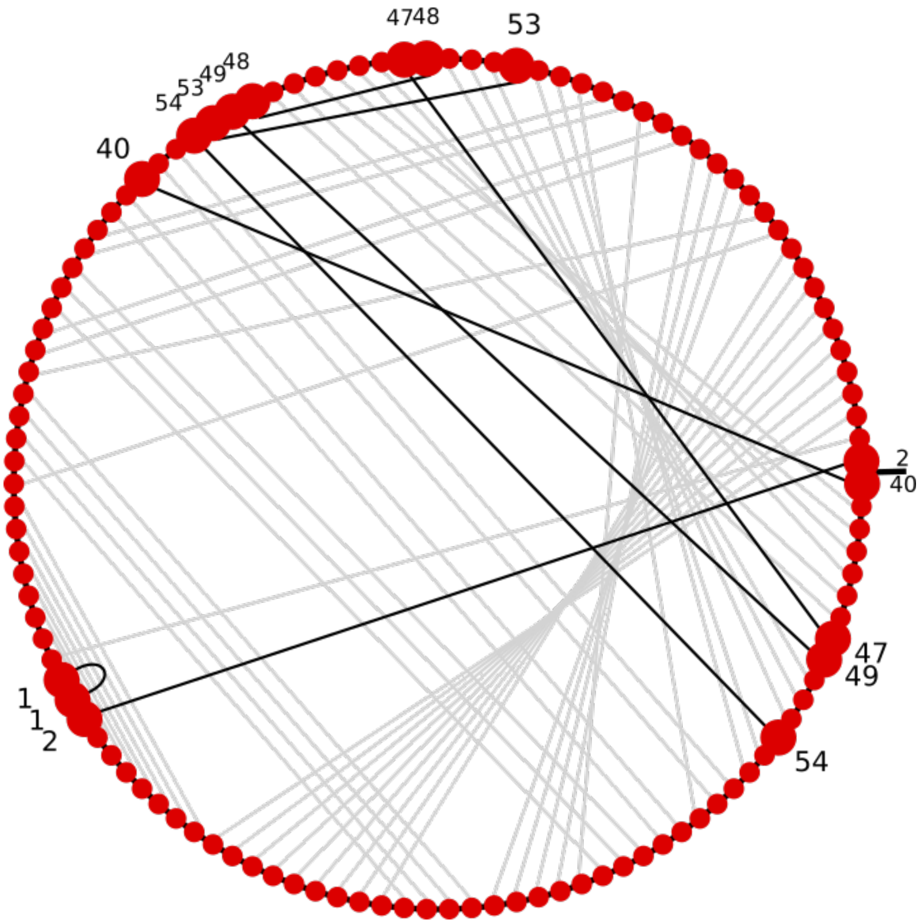

Chord diagram for contig6325.0.0
Black tick mark indicates the start of the scrambled pointer list (reading clockwise). Grey lines indicate pointers in repeat/return words. Black lines indicate the pointers remaining after iterative removal of odd-even patterns (repeat and return words). To improve visibility, only those pointers remaining are marked with numbers.

contig17650.0

MDSs found in MIC locus ctg68245

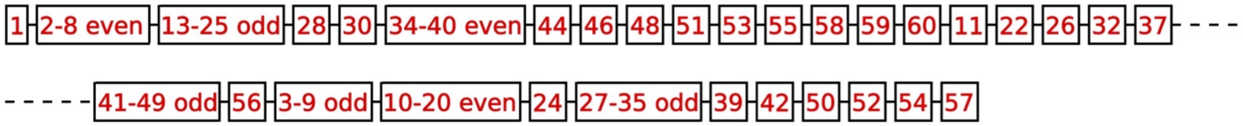

Linear representation of MDSs in the MIC locus.

Pointer list: [1, 2, 3, 4, 5, 6, 7, 10, 11, 12, 13, 14, 15, 16, 17, 18, 19, 20, 21, 22, 23, 25, 26, 27, 28, 31, 32, 33, 34, 35, 36, 37, 38, 41, 42, 43, 44, 45, 46, 48, 49, 50, 51, 52, 53, 55, 8, 9, 19, 20, 23, 24, 29, 30, 34, 35, 38, 39, 40, 41, 42, 43, 44, 45, 46, 47, 53, 54, 1, 2, 3, 4, 5, 6, 7, 8, 9, 10, 11, 12, 13, 14, 15, 16, 17, 18, 21, 22, 24, 25, 26, 27, 28, 29, 30, 31, 32, 33, 36, 37, 39, 40, 47, 48, 49, 50, 51, 52, 54, 55]

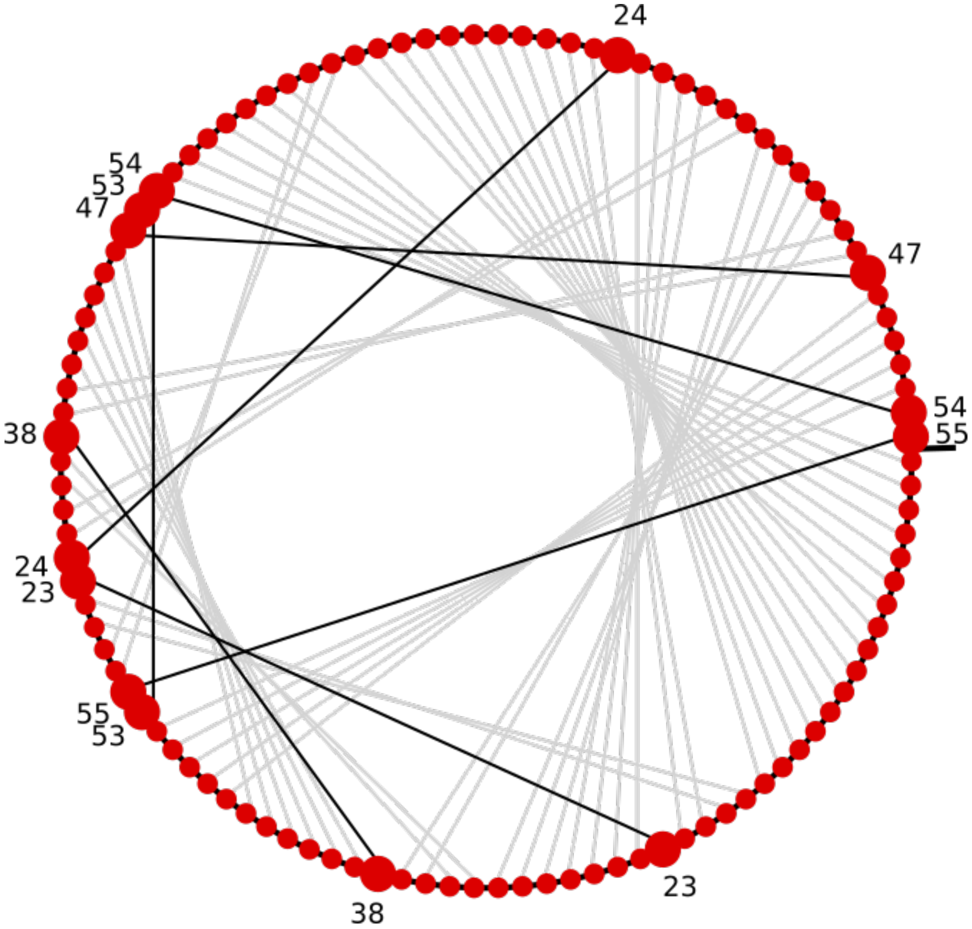

Chord diagram for contig17650.0

Black tick mark indicates the start of the scrambled pointer list (reading clockwise). Grey lines indicate pointers in repeat/return words. Black lines indicate the pointers remaining after iterative removal of odd-even patterns (repeat and return words). To improve visibility, only those pointers remaining are marked with numbers.

contig19385.0

MDSs found in MIC locus ctg67907

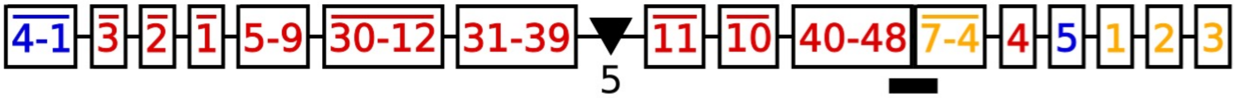

Linear representation of MDSs in the MIC locus. Red: contig19385.0; blue: contig3791.0; yellow: contig11385.0. The triangle corresponds to the 5 MDSs of the nonscrambled contig9517.0.

Pointer list: [1, 2, 3, 5, 4, 5, 6, 4, 3, 6, 1, 2]

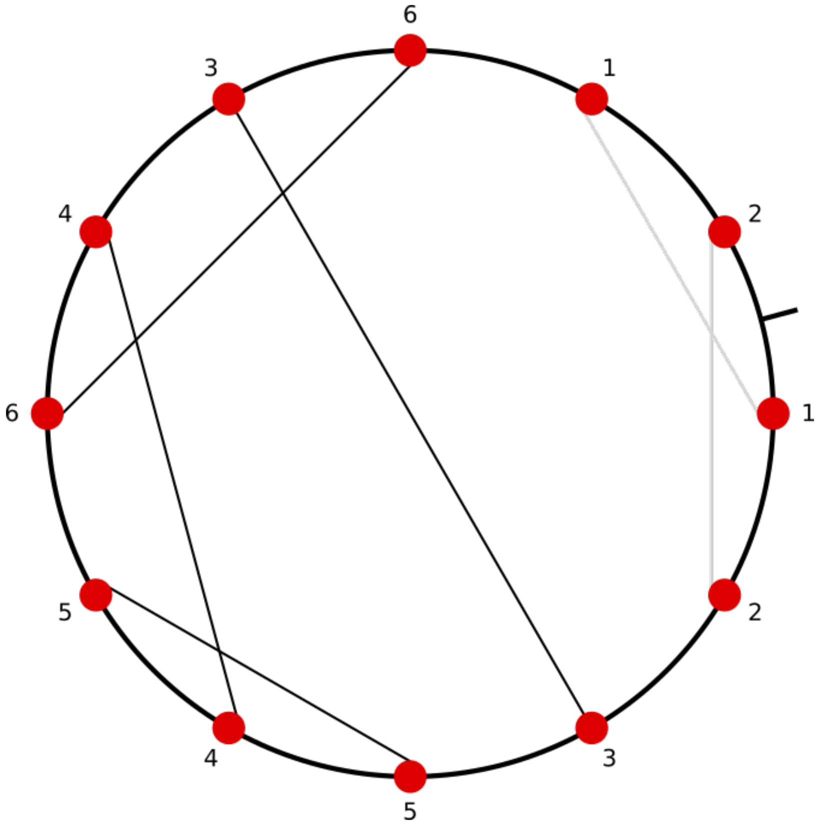

Chord diagram for contig19385.0

Black tick mark indicates the start of the scrambled pointer list (reading clockwise). Grey lines indicate pointers in repeat/return words. Black lines indicate the pointers remaining after iterative removal of odd-even patterns (repeat and return words).

contig7885.0

MDSs found in MIC locus ctg76979

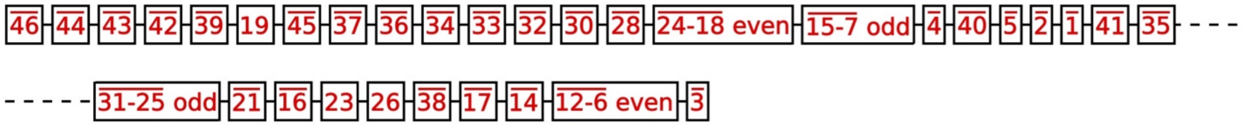

Linear representation of MDSs in the MIC locus.

Pointer list: [39, 38, 37, 35, 34, 17, 18, 39, 38, 33, 32, 31, 30, 29, 28, 27, 26, 23, 22, 21, 20, 19, 18, 17, 16, 14, 13, 12, 11, 10, 9, 8, 7, 6, 5, 3, 2, 36, 35, 4, 3, 1, 37, 36, 32, 31, 30, 29, 28, 27, 26, 25, 24, 23, 20, 19, 15, 14, 21, 22, 24, 25, 34, 33, 16, 15, 13, 12, 11, 10, 9, 8, 7, 6, 5, 4, 2, 1]

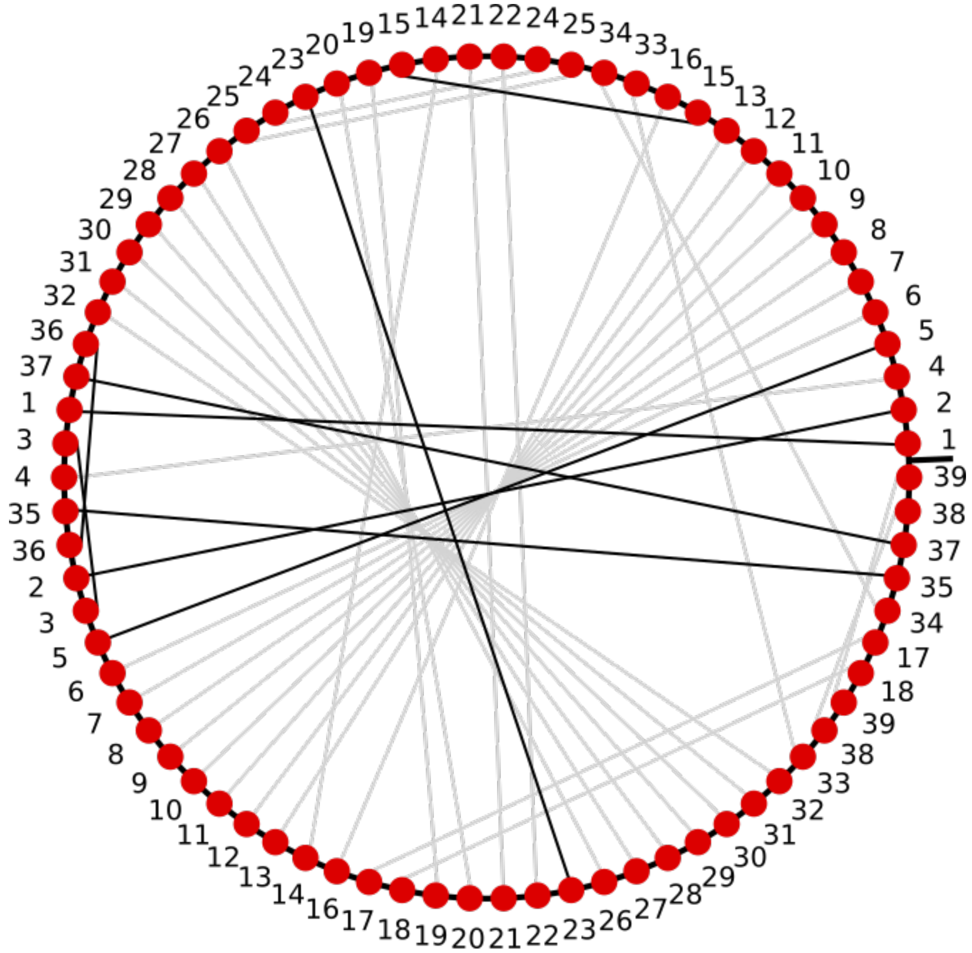

Chord diagram for contig7885.0

Black tick mark indicates the start of the scrambled pointer list (reading clockwise). Grey lines indicate pointers in repeat/return words. Black lines indicate the pointers remaining after iterative removal of odd-even patterns (repeat and return words).

contig14667.0

MDSs found in MIC locus ctg70616

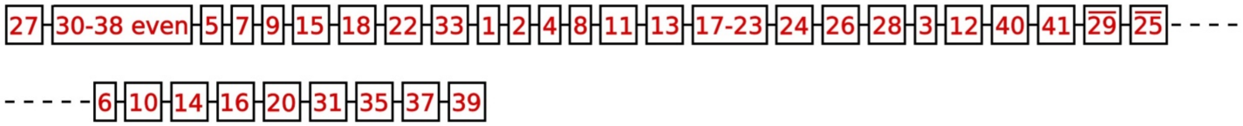

Linear representation of MDSs in the MIC locus.

Pointer list: [24, 25, 27, 28, 29, 30, 31, 32, 33, 34, 35, 36, 3, 4, 5, 6, 7, 8, 13, 14, 16, 17, 20, 21, 30, 31, 1, 2, 3, 6, 7, 9, 10, 11, 12, 15, 16, 17, 18, 19, 20, 21, 22, 23, 24, 25, 26, 1, 2, 10, 11, 37, 27, 26, 23, 22, 4, 5, 8, 9, 12, 13, 14, 15, 18, 19, 28, 29, 32, 33, 34, 35, 36, 37]

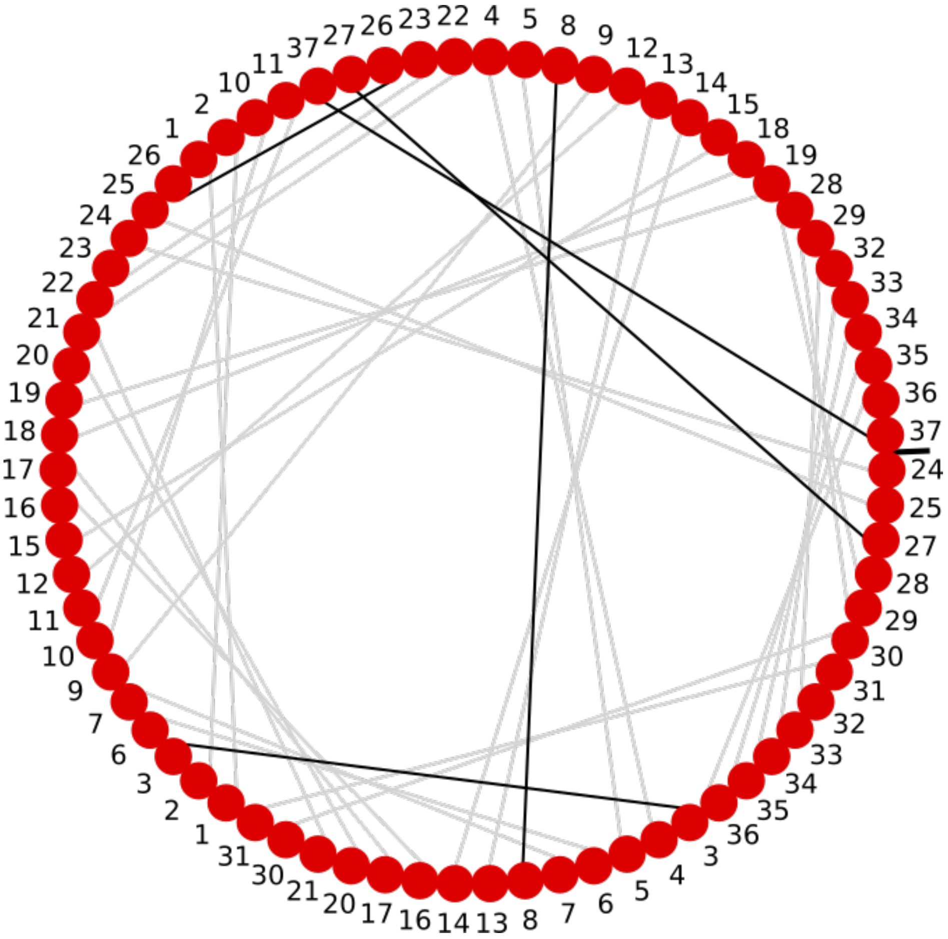

Chord diagram for contig14667.0

Black tick mark indicates the start of the scrambled pointer list (reading clockwise). Grey lines indicate pointers in repeat/return words. Black lines indicate the pointers remaining after iterative removal of odd-even patterns (repeat and return words).

contig101.0

MDSs found in MIC locus ctg87805

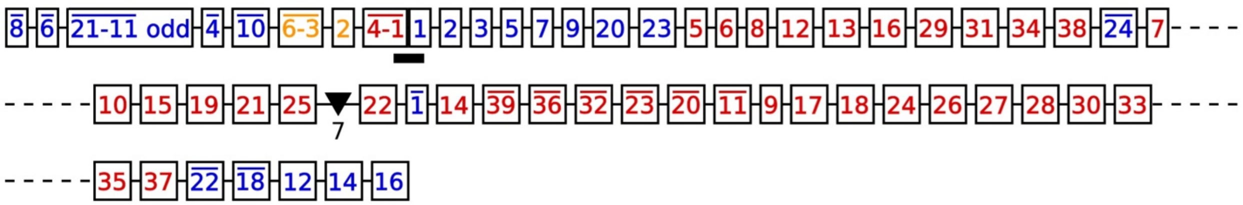

Linear representation of MDS in the MIC locus. Red: contig101.0; blue: contig9427.0; yellow: contig4977.0. The triangle corresponds to the 7 MDSs of the nonscrambled contig20796.0.

Pointer list: [1, 1, 2, 3, 4, 7, 8, 10, 11, 20, 21, 22, 23, 25, 26, 29, 30, 2, 3, 5, 6, 9, 10, 12, 13, 14, 15, 18, 19, 15, 16, 8, 9, 30, 28, 27, 24, 23, 17, 16, 14, 13, 7, 6, 4, 5, 11, 12, 17, 18, 19, 20, 21, 22, 24, 25, 26, 27, 28, 29]

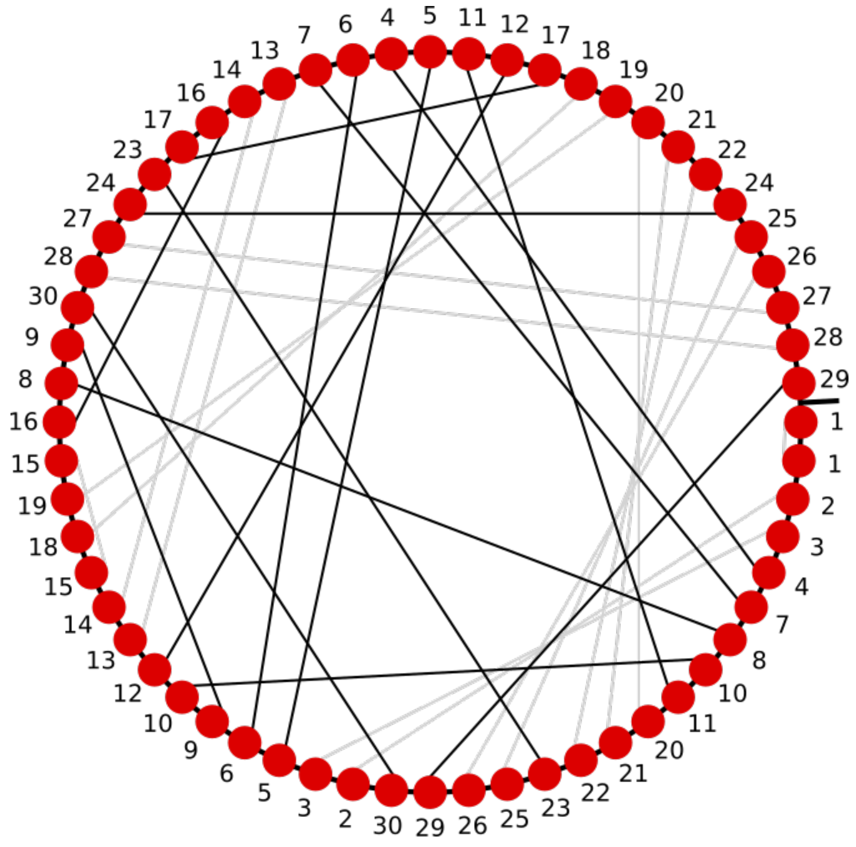

Chord diagram for contig101.0.
Black tick mark indicates the start of the scrambled pointer list (reading clockwise). Grey lines indicate pointers in repeat/return words. Black lines indicate the pointers remaining after iterative removal of odd-even patterns (repeat and return words).

contig21044.0

MDSs found in MIC locus ctg67570

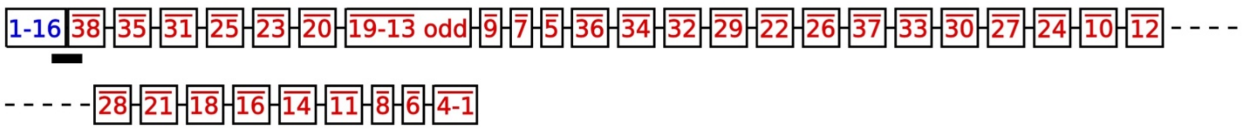

Linear representation of MDSs in the MIC locus. Red: contig21044.0; blue: contig20875.0.

Pointer list: [33, 31, 30, 27, 26, 21, 20, 19, 18, 16, 15, 14, 13, 12, 11, 10, 9, 6, 5, 4, 3, 2, 1, 32, 31, 30, 29, 28, 27, 25, 24, 18, 17, 22, 21, 33, 32, 29, 28, 26, 25, 23, 22, 20, 19, 7, 6, 9, 8, 24, 23, 17, 16, 15, 14, 13, 12, 11, 10, 8, 7, 5, 4, 3, 2, 1]

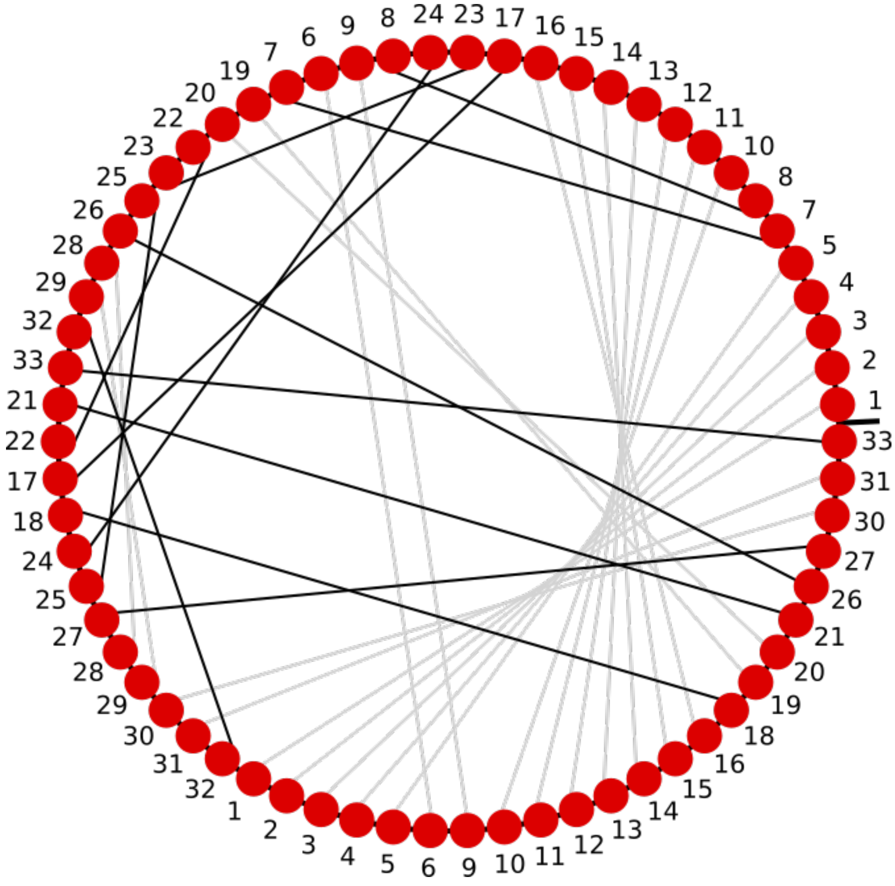

Chord diagram for contig21044.0

Black tick mark indicates the start of the scrambled pointer list (reading clockwise). Grey lines indicate pointers in repeat/return words. Black lines indicate the pointers remaining after iterative removal of odd-even patterns (repeat and return words).

contig9447.0

MDSs found in MIC locus ctg78920

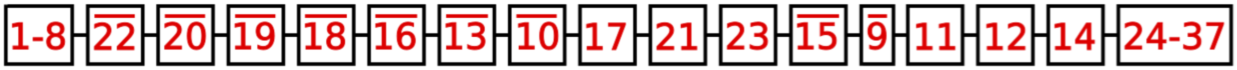

Linear representation of MDSs in the MIC locus.

Pointer list: [1, 12, 11, 10, 9, 8, 7, 5, 4, 3, 2, 8, 9, 10, 11, 12, 13, 7, 6, 2, 1, 3, 4, 5, 6, 13]

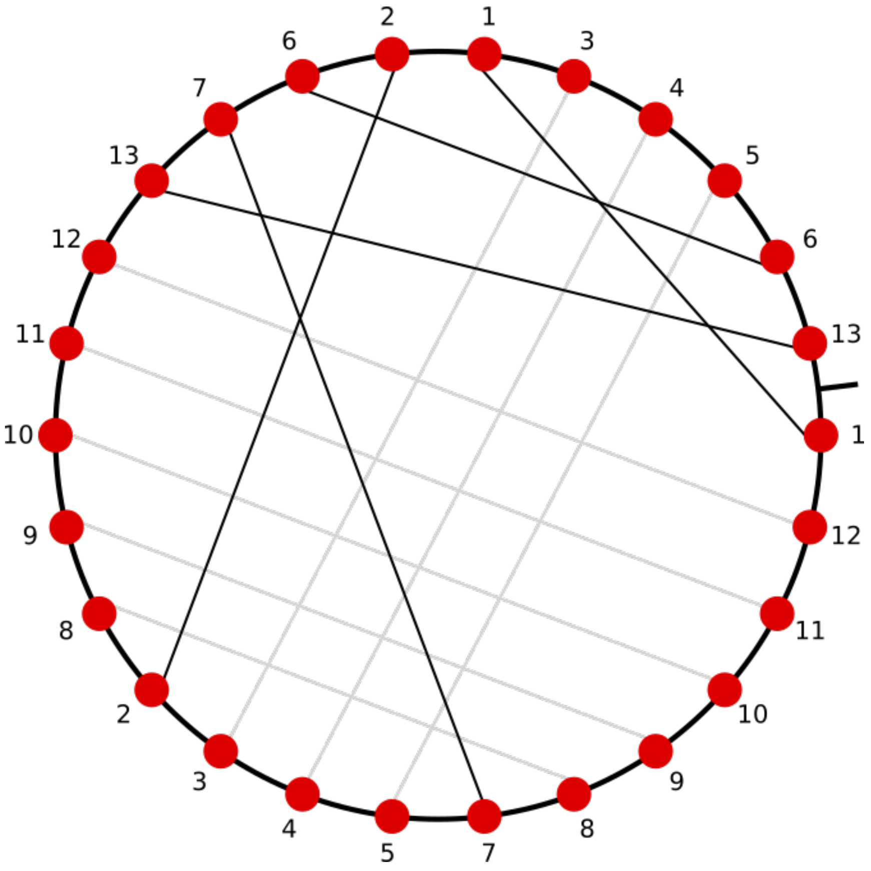

Chord diagram for contig9447.0
Black tick mark indicates the start of the scrambled pointer list (reading clockwise). Grey lines indicate pointers in repeat/return words. Black lines indicate the pointers remaining after iterative removal of odd-even patterns (repeat and return words).

contig14762.0

MDSs found in MIC locus ctg88985

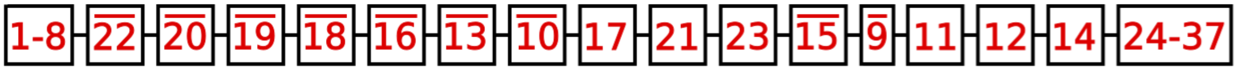

Linear representation of MDSs in the MIC locus.

Pointer list: [2, 3, 6, 7, 9, 10, 12, 13, 14, 15, 16, 17, 18, 19, 21, 22, 25, 26, 27, 28, 29, 30, 31, 32, 33, 34, 35, 36, 1, 2, 3, 4, 5, 6, 7, 8, 10, 11, 17, 18, 20, 21, 22, 23, 24, 25, 28, 29, 30, 31, 32, 33, 34, 35, 36, 1, 4, 5, 8, 9, 11, 12, 13, 14, 15, 16, 19, 20, 23, 24, 26, 27]

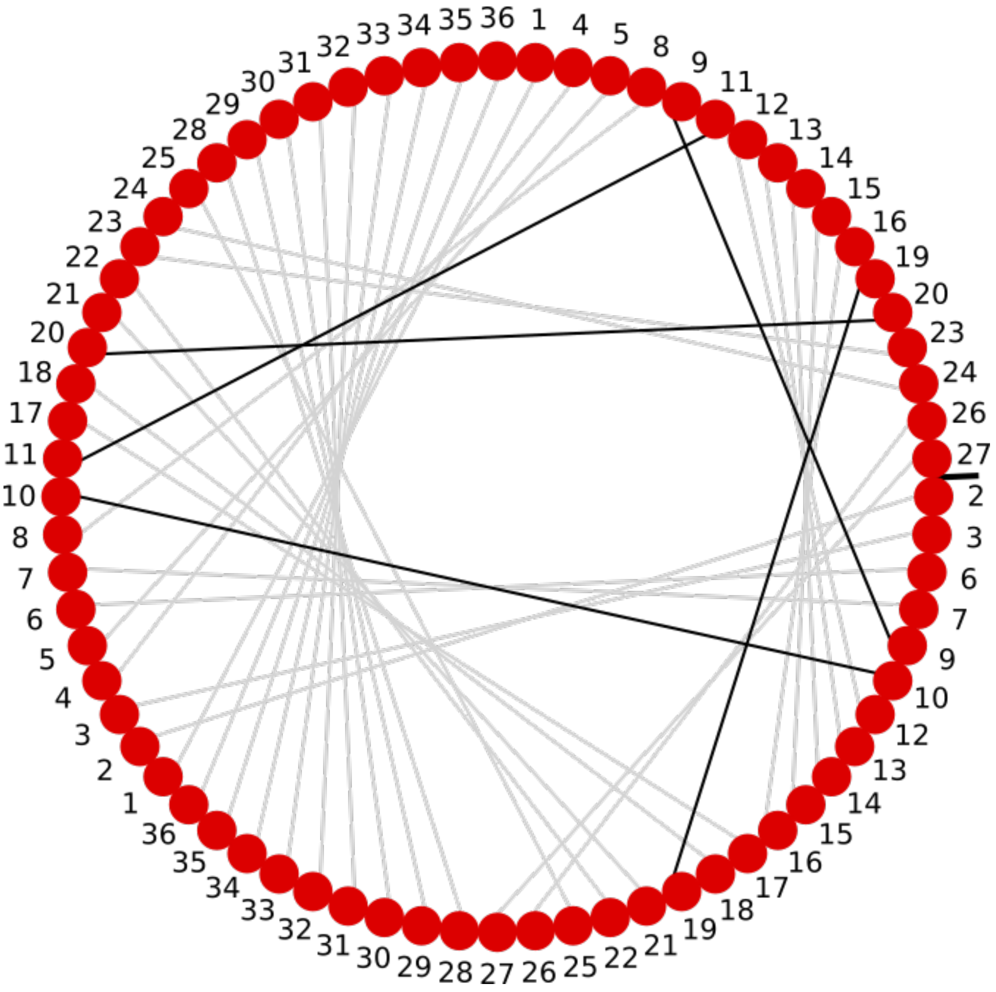

Chord diagram for contig14762.0

Black tick mark indicates the start of the scrambled pointer list (reading clockwise). Grey lines indicate pointers in repeat/return words. Black lines indicate the pointers remaining after iterative removal of odd-even patterns (repeat and return words).

contig1443.1

MDSs found in MIC locus ctg68905

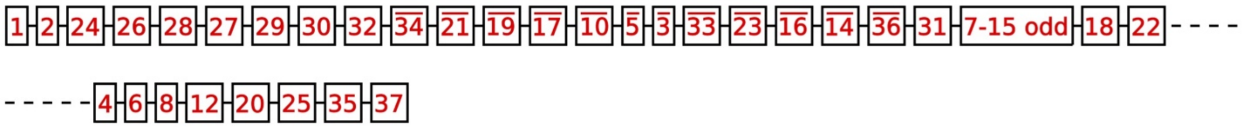

Linear representation of MDSs in the MIC locus.

Pointer list: [1, 22, 23, 24, 25, 26, 27, 25, 26, 27, 28, 29, 30, 32, 31, 20, 19, 18, 17, 16, 15, 9, 8, 4, 3, 2, 1, 31, 30, 22, 21, 15, 14, 13, 12, 34, 33, 28, 29, 5, 6, 7, 8, 9, 10, 11, 12, 13, 14, 16, 17, 20, 21, 2, 3, 4, 5, 6, 7, 10, 11, 18, 19, 23, 24, 32, 33, 34]

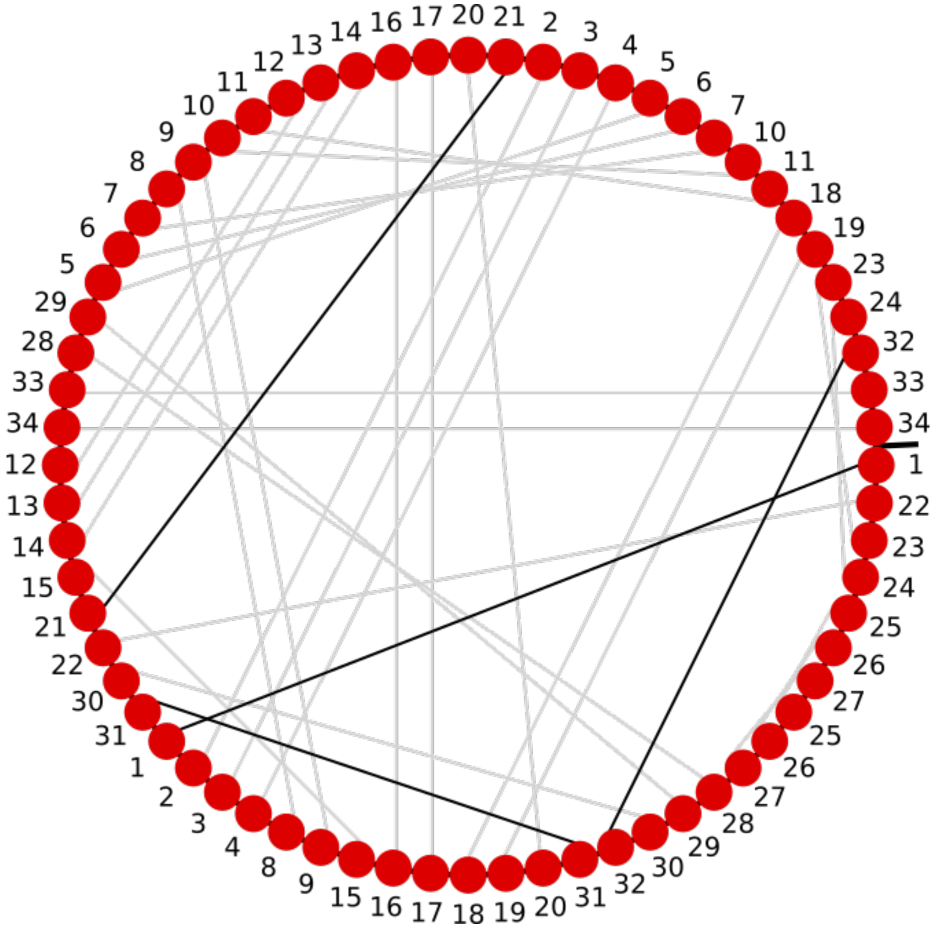

Chord diagram for contig1443.1
Black tick mark indicates the start of the scrambled pointer list (reading clockwise). Grey lines indicate pointers in repeat/return words. Black lines indicate the pointers remaining after iterative removal of odd-even patterns (repeat and return words).

contig13832.0

MDSs found in MIC locus ctg72050

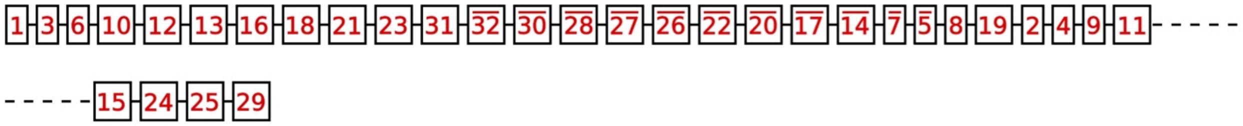

Linear representation of MDSs in the MIC locus.

Pointer list: [1, 2, 3, 5, 6, 9, 10, 11, 12, 14, 15, 16, 17, 19, 20, 21, 22, 26, 27, 27, 26, 25, 24, 23, 21, 20, 19, 18, 16, 15, 13, 12, 7, 6, 5, 4, 7, 8, 17, 18, 1, 2, 3, 4, 8, 9, 10, 11, 13, 14, 22, 23, 24, 25]

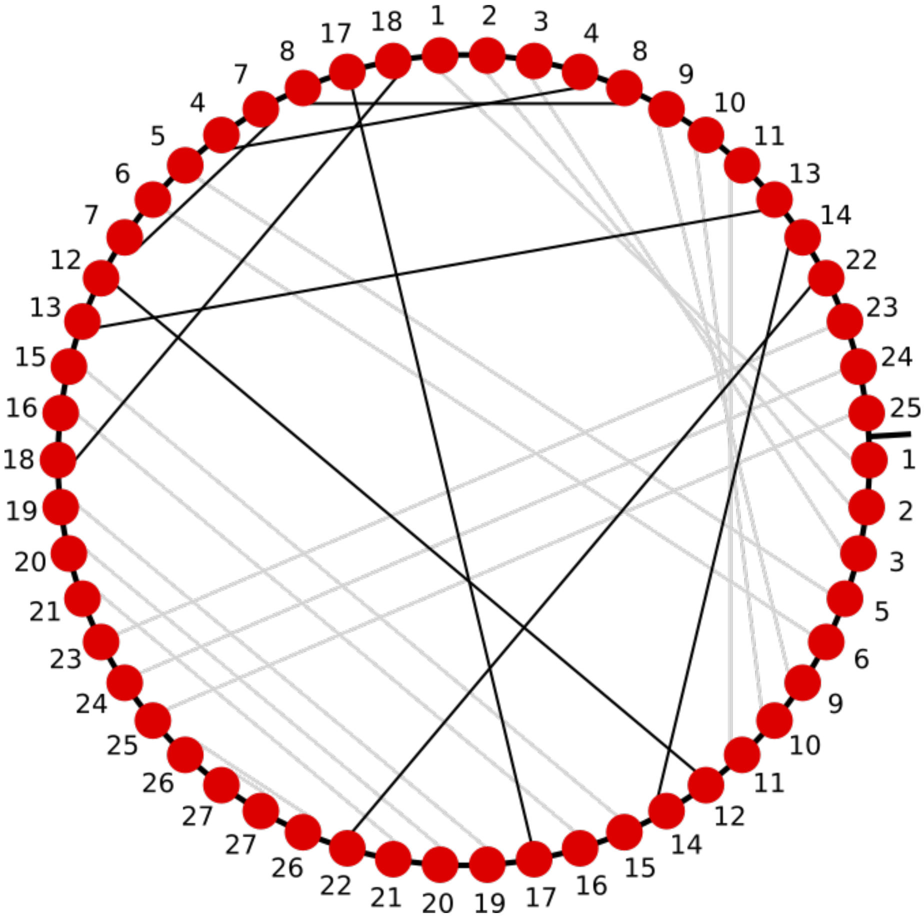

Chord diagram for contig13832.0
Black tick mark indicates the start of the scrambled pointer list (reading clockwise). Grey lines indicate pointers in repeat/return words. Black lines indicate the pointers remaining after iterative removal of odd-even patterns (repeat and return words).

contig10305.0

MDSs found in MIC locus ctg68368

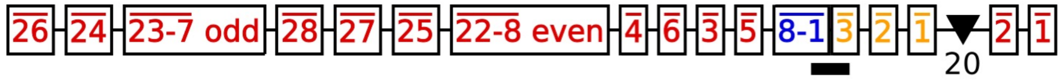

Linear representation of MDSs in the MIC locus. Red: contig10305.0; blue: contig19660.0; yellow: contig12781.0. The triangle corresponds to the nonscrambled contigs 19665.0 and 19141.0 with 6 and 14 MDSs, respectively.

Pointer list: [24, 23, 22, 21, 20, 19, 18, 17, 16, 15, 14, 13, 12, 11, 10, 9, 8, 7, 6, 5, 24, 23, 22, 21, 20, 19, 18, 17, 16, 15, 14, 13, 12, 11, 10, 9, 8, 7, 6, 3, 2, 5, 4, 2, 1, 4, 3, 1]

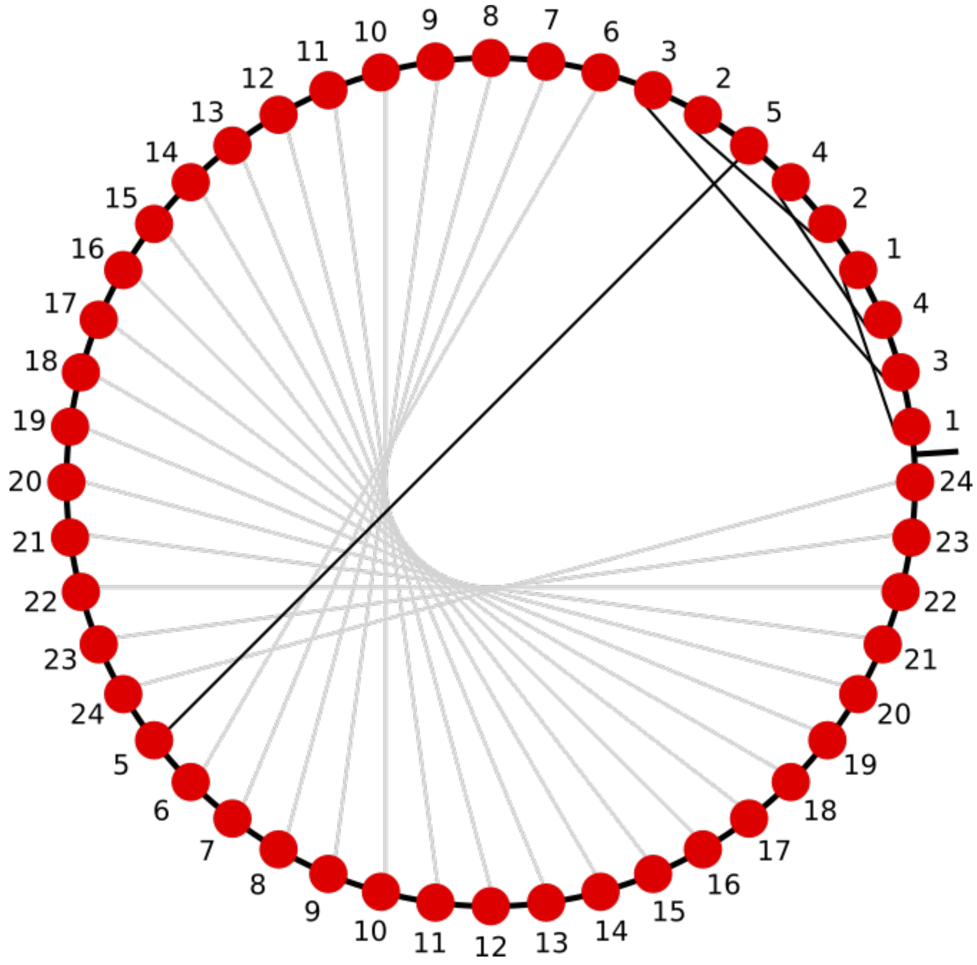

Chord diagram for contig10305.0

Black tick mark indicates the start of the scrambled pointer list (reading clockwise). Grey lines indicate pointers in repeat/return words. Black lines indicate the pointers remaining after iterative removal of odd-even patterns (repeat and return words).

contig582.1

MDSs found in MIC locus ctg67690

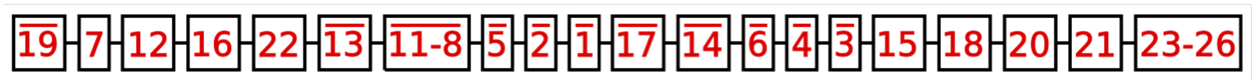

Linear representation of MDSs in the MIC locus.

Pointer list: [14, 13, 4, 5, 6, 7, 10, 11, 15, 16, 8, 7, 6, 5, 3, 2, 1, 12, 11, 9, 8, 4, 3, 2, 1, 9, 10, 12, 13, 14, 15, 16]

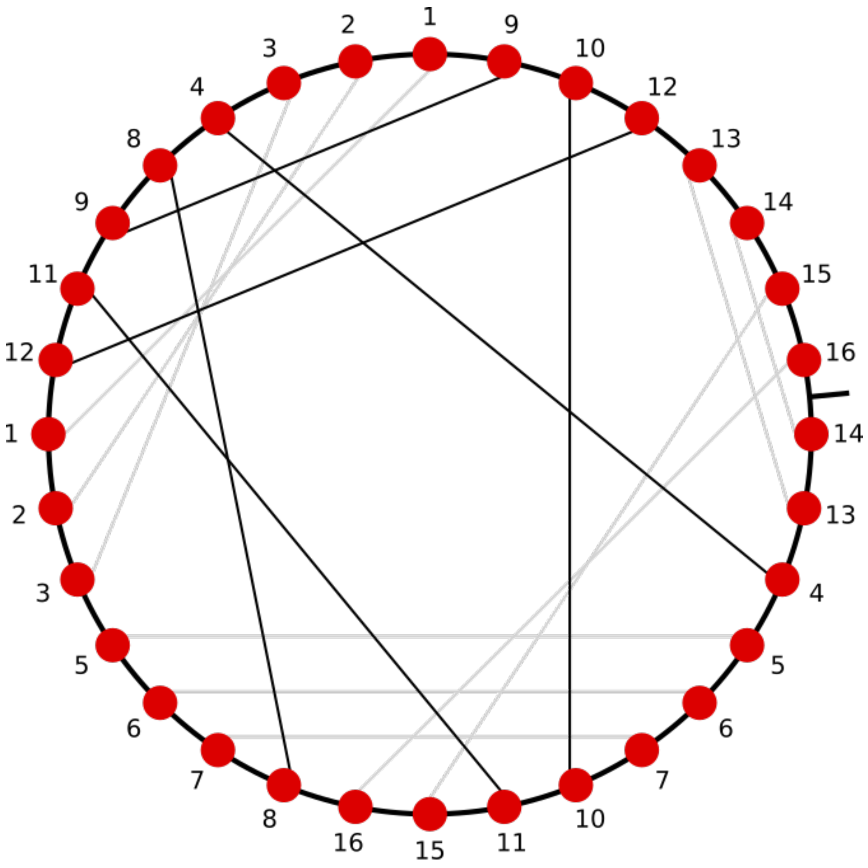

Chord diagram for contig582.1
Black tick mark indicates the start of the scrambled pointer list (reading clockwise). Grey lines indicate pointers in repeat/return words. Black lines indicate the pointers remaining after iterative removal of odd-even patterns (repeat and return words).

contig9655.0

MDSs found in MIC locus ctg67180

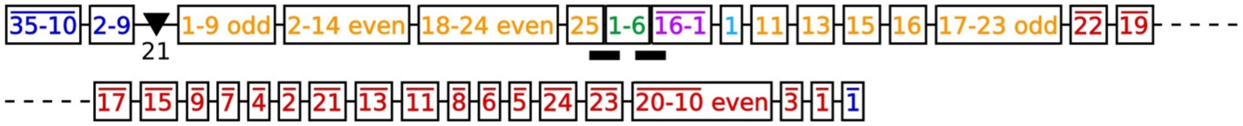

Linear representation of MDSs in the MIC locus. Red: contig9655.0; blue: contig15801.0; yellow: contig11026.0; green: contig14040.0; purple: contig14353.0; light blue: contig12848.0. The triangle corresponds to the nonscrambled contigs 18706.0 and 14941.0 with 9 and 12 MDSs, respectively.

Pointer list: [21, 20, 18, 17, 16, 15, 14, 13, 8, 7, 6, 5, 4, 3, 2, 1, 20, 19, 12, 11, 10, 9, 7, 6, 5, 4, 21, 19, 18, 17, 16, 15, 14, 13, 12, 11, 10, 9, 8, 3, 2, 1]

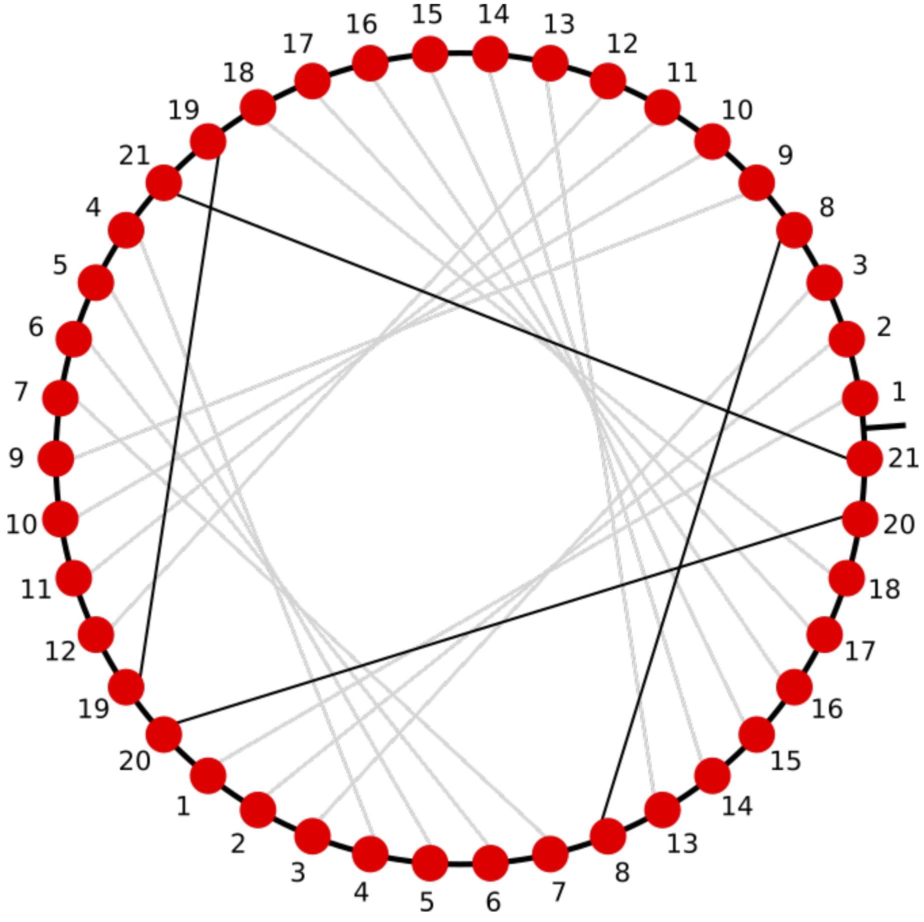

Chord diagram for contig9655.0
Black tick mark indicates the start of the scrambled pointer list (reading clockwise). Grey lines indicate pointers in repeat/return words. Black lines indicate the pointers remaining after iterative removal of odd-even patterns (repeat and return words).

contig18067.0

MDSs found in MIC locus ctg70389

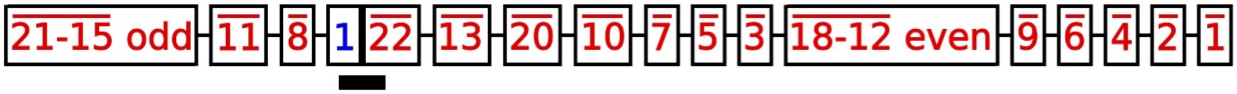

Linear representation of MDSs in the MIC locus. Red: contig18067.0; blue: contig16133.0.

Pointer list: [20, 19, 18, 17, 16, 15, 14, 13, 10, 9, 7, 6, 20, 12, 11, 19, 18, 9, 8, 6, 5, 4, 3, 2, 1, 17, 16, 15, 14, 13, 12, 11, 10, 8, 7, 5, 4, 3, 2, 1]

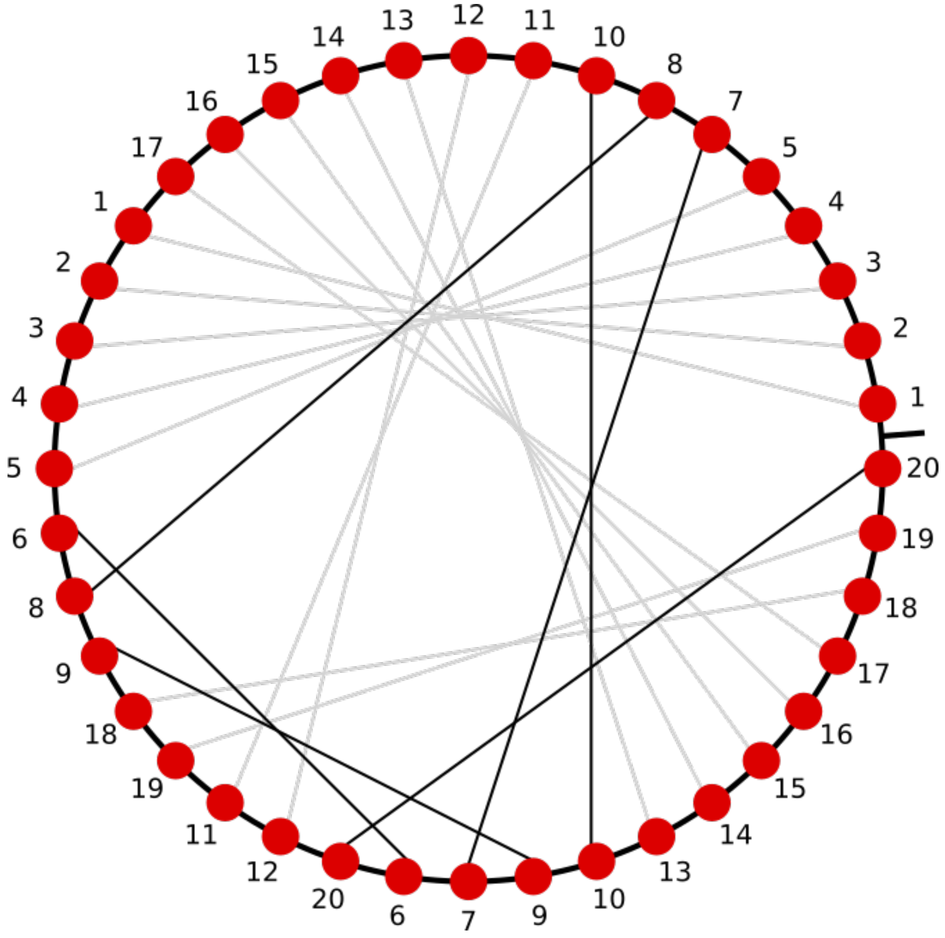

Chord diagram for contig18067.0

Black tick mark indicates the start of the scrambled pointer list (reading clockwise). Grey lines indicate pointers in repeat/return words. Black lines indicate the pointers remaining after iterative removal of odd-even patterns (repeat and return words).

contig9889.0

MDSs found in MIC locus ctg68483

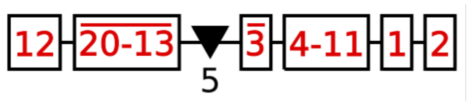

Linear representation of MDSs in the MIC locus. Red: contig9889.0. The triangle corresponds to the 5 MDSs of the nonscrambled contig20935.

Pointer list: [3, 4, 4, 2, 1, 2, 3, 1]

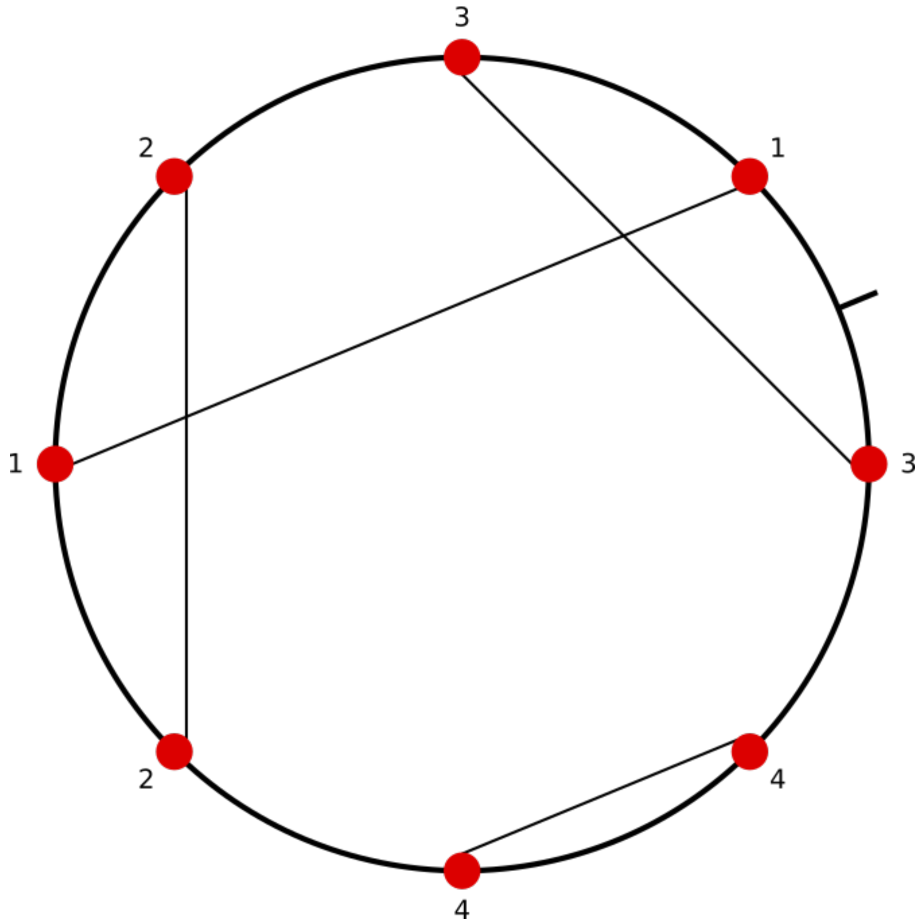

Chord diagram for contig9889.0
Black tick mark indicates the start of the scrambled pointer list (reading clockwise).
Black lines indicate scrambled pointers.

contig6570.0

MDSs found in MIC locus ctg68482

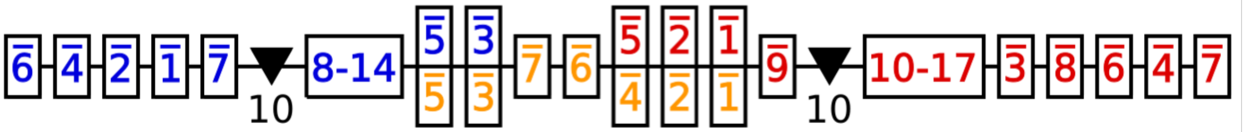

Linear representation of MDSs in the MIC locus. Red: contig6570.0; blue: contig9440.0; yellow: contig8220.0. The first triangle corresponds to the 10 MDSs of the nonscrambled contig1183.0 and the second triangle to the 10 MDSs of the nonscrambled contig5057.0.

Pointer list: [4, 3, 1, 8, 7, 8, 2, 1, 7, 6, 5, 4, 3, 2, 6, 5]

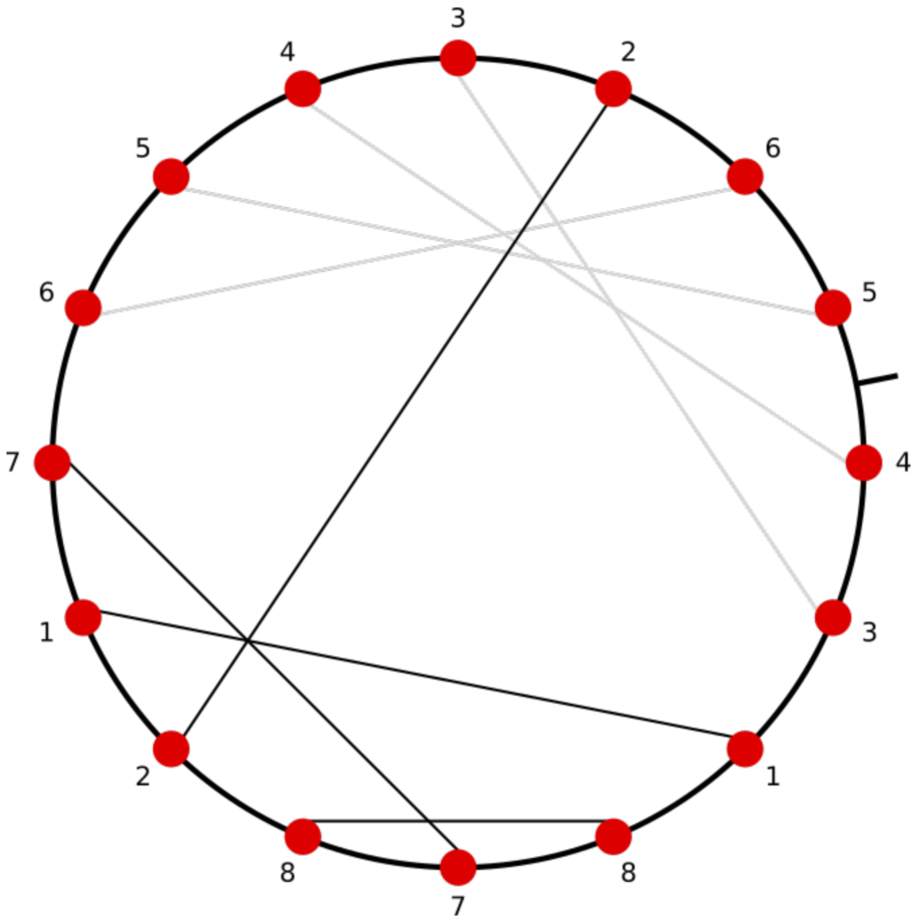

Chord diagram for contig6570.0

Black tick mark indicates the start of the scrambled pointer list (reading clockwise). Grey lines indicate pointers in repeat/return words. Black lines indicate the pointers remaining after iterative removal of odd-even patterns (repeat and return words).

contig8380.0

MDSs found in MIC locus ctg67241

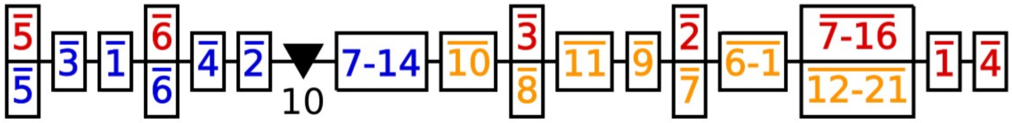

Linear representation of MDSs in the MIC locus. Red: contig8380.0; blue: contig19700.0; yellow: contig918.1. The triangle corresponds to the 10 MDSs of the nonscrambled contig17042.0.

Pointer list: [4, 3, 5, 4, 2, 1, 5, 1, 3, 2]

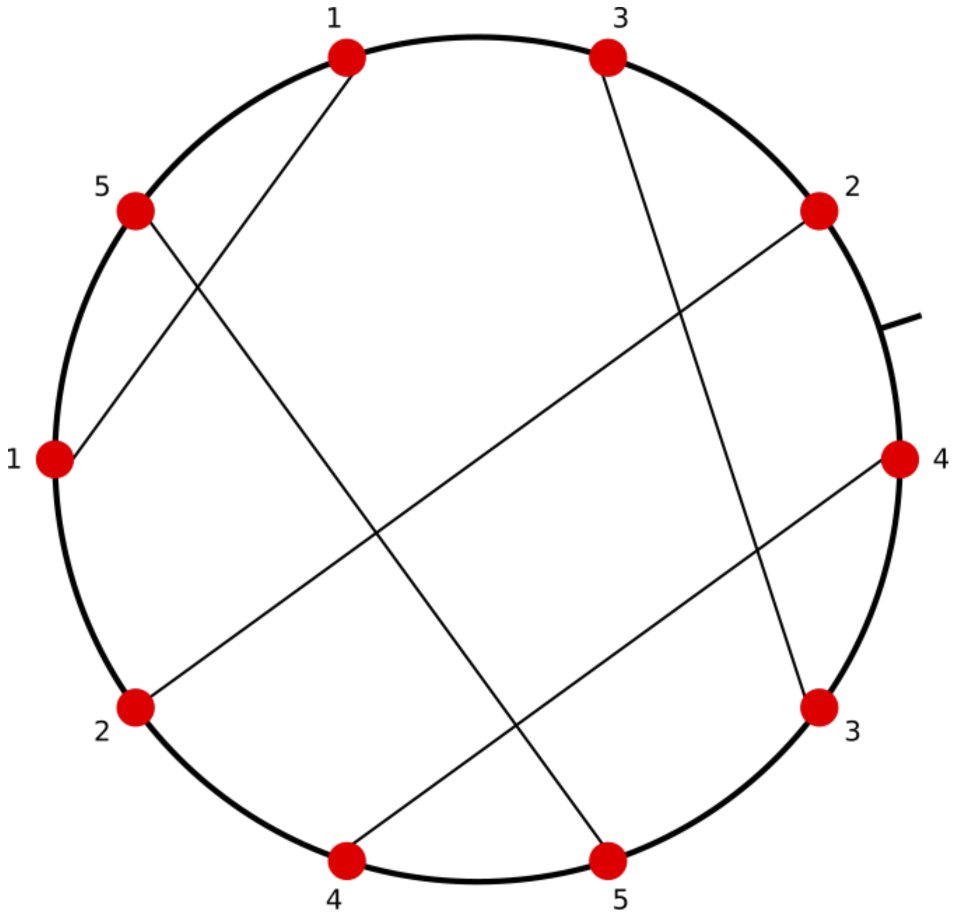

Chord diagram for contig8380.0
Black tick mark indicates the start of the scrambled pointer list (reading clockwise).
Black lines indicate scrambled pointers.

contig1679.0

MDSs found in MIC locus ctg67241

2 - 3 - $\bar{8}$ - $\bar{7}$ - $\bar{3}$ - $\bar{2}$ - $\bar{1}$ - $\bar{1}$ - $\bar{4}$ - 5 - 6

Linear representation of MDSs in the MIC locus. Red: contig1679.0; blue: contig6374.0.

Pointer list: [1, 2, 4, 1, 3, 2, 3, 4]

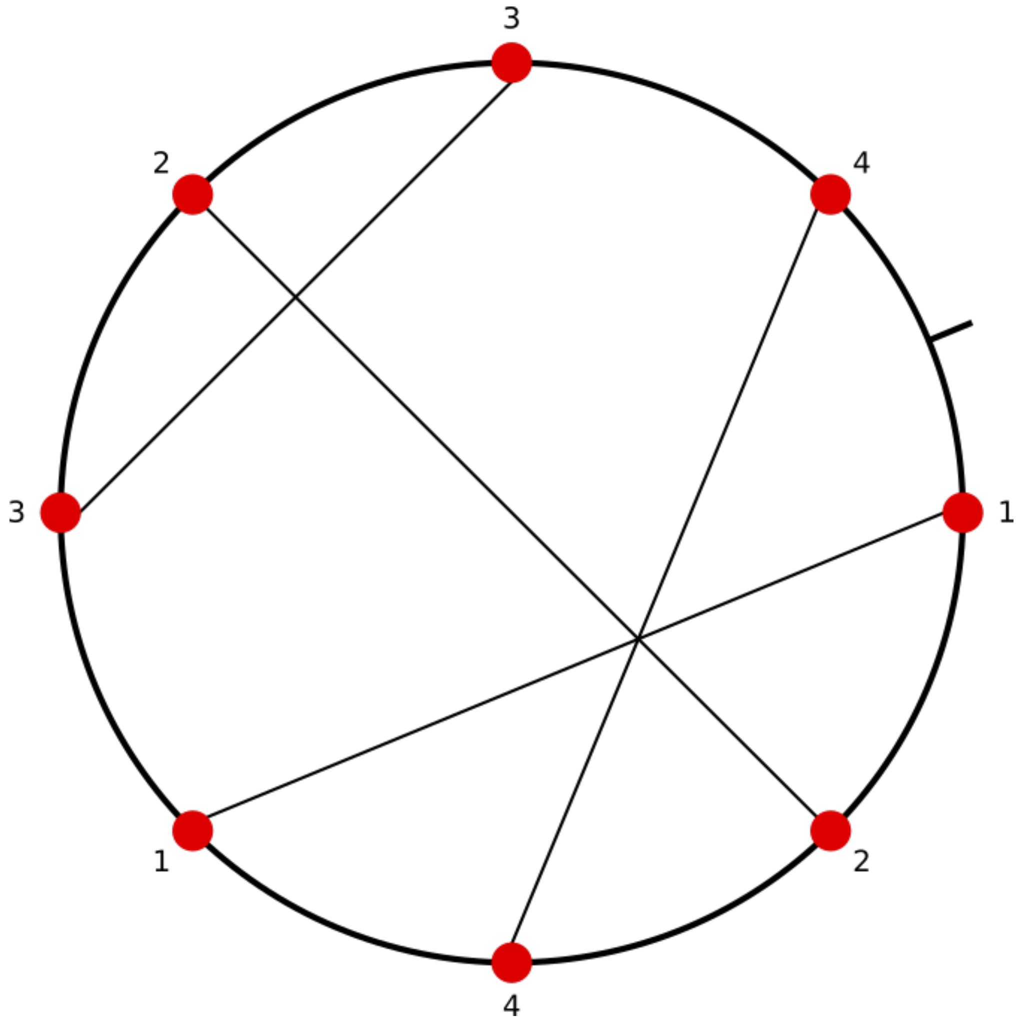

Chord diagram for contig1679.0
Black tick mark indicates the start of the scrambled pointer list (reading clockwise).
Black lines indicate scrambled pointers.
